# Effect of acupuncture inclusion in the enhanced recovery after surgery protocol on tumor patient gastrointestinal function: a systematic review and meta-analysis of randomized controlled studies

**DOI:** 10.3389/fonc.2023.1232754

**Published:** 2023-08-15

**Authors:** Jiu Chen, Tianxiao Fu, Li Liu, Yirui Xie, Youdi Li

**Affiliations:** ^1^ Department of Traditional Chinese Medicine, The First Affiliated Hospital, School of Medicine, Zhejiang University, Hangzhou, China; ^2^ Department of Library, The First Affiliated Hospital, School of Medicine, Zhejiang University, Hangzhou, China; ^3^ State Key Laboratory for Diagnosis and Treatment of Infectious Diseases, National Clinical Research Center for Infectious Diseases, Collaborative Innovation Center for Diagnosis and Treatment of Infectious Diseases, Hangzhou, China; ^4^ The Department of Infectious Diseases, The First Affiliated Hospital, School of Medicine, Zhejiang University, Hangzhou, China

**Keywords:** acupuncture, tumor, enhanced recovery after surgery, meta-analysis, gastrointestinal function

## Abstract

**Introduction:**

Acupuncture has been shown to be effective in restoring gastrointestinal function in tumor patients receiving the enhanced recovery after surgery (ERAS) protocol. The present systematic review and meta-analysis aimed to evaluate the rationality and efficacy of integrating acupuncture in the ERAS strategy to recuperate gastrointestinal function.

**Methods:**

We searched eleven databases for relevant randomized clinical trials (RCTs) of acupuncture for the treatment of gastrointestinal dysfunction in tumor patients treated with the ERAS protocol. The quality of each article was assessed using the Cochrane Collaboration risk of bias criteria and the modified Jadad Scale. As individual symptoms, the primary outcomes were time to postoperative oral food intake, time to first flatus, time to first distension and peristaltic sound recovery time (PSRT). Pain control, adverse events, and acupoint names reported in the included studies were also investigated.

**Results:**

Of the 211 reviewed abstracts, 9 studies (702 patients) met eligibility criteria and were included in the present systematic review and meta‑analysis. Compared to control groups, acupuncture groups showed a significant reduction in time to postoperative oral food intake [standardized mean difference (SMD) = -0.77, 95% confidence interval (CI) -1.18 to -0.35], time to first flatus (SMD=-0.81, 95% CI -1.13 to -0.48), time to first defecation (SMD=-0.91, 95% CI -1.41 to -0.41, PSRT (SMD=-0.92, 95% CI -1.93 to 0.08), and pain intensity (SMD=-0.60, 95% CI -0.83 to -0.37).The Zusanli (ST36) and Shangjuxu (ST37) acupoints were used in eight of the nine included studies. Adverse events related to acupuncture were observed in two studies, and only one case of bruising was reported.

**Discussion:**

The present systematic review and meta‑analysis suggested that acupuncture significantly improves recovery of gastrointestinal function and pain control in tumor patients receiving the ERAS protocol compared to the control group. Moreover, ST36 and ST37 were the most frequently used acupoints. Although the safety of acupuncture was poorly described in the included studies, the available data suggested that acupuncture is a safe treatment with only mild side effects. These findings provide evidence-based recommendations for the inclusion of acupuncture in the ERAS protocol for tumor patients.

**Systematic review registration:**

https://www.crd.york.ac.uk/prospero/ PROSPERO, identifier CRD42023430211.

## 1 Introduction

Cancer is a major worldwide problem, and surgery continues to be the mainstay treatment for tumors (1). The enhanced recovery after surgery (ERAS) protocol is a novel surgical strategy that consists of multimodal, multidisciplinary perioperative protocols aimed at achieving optimal patient outcomes (2). As numerous clinical studies have confirmed its effectiveness in improving recovery after surgery, the ERAS protocol is now used in most tumor surgical subspecialties (3, 4).

Gastrointestinal dysfunction has long been a challenging clinical problem for patients using both the ERAS protocol and conventional strategies (5). Although dysfunction is generally due to multifactorial mechanisms, surgical stress and associated autonomic, neuroendocrine, and immune responses play a key pathophysiological role (6), and the stress and responses are more serious for tumor patients subjected to ERAS strategies due to the more aggressive surgical methods for preventing recurrence and metastasis. Moreover, these responses that lead to gastrointestinal dysfunction may be unavoidable and may not be completely resolved for a long time after surgery (6–8). ERAS strategy-related gastrointestinal dysfunction in the state of sufficient analgesia is under increasing scrutiny, especially in elderly and pediatric patients (9). Because non-narcotic pain control agents are considered essential to the success of the ERAS strategy (9), acetaminophen, non-steroidal anti-inflammatory drugs (NSAIDs), and other analgesics are increasingly used to improve pain control (10). However, analgesics should be administered with caution as these drugs are associated with gastrointestinal side effects and exacerbate existing gastrointestinal dysfunction, which can lead to serious complications, such as gastrointestinal bleeding and anastomotic leaks (9, 11–13). In patients undergoing abdominal surgery, this problem is particularly pronounced due to the presence of underlying diseases, which may explain the conclusion in a previous meta-analysis that 30-day readmission rates significantly increase rather than decrease after upper gastrointestinal tract surgery in patients treated with the ERAS strategy compared to patients using a conventional surgical strategy (14). Treatment options for restoring gastrointestinal function are limited (9). The use of chewing gum as a sham feeding has been shown to be a safe and effective intervention for reducing the incidence of ileus after colorectal surgery (15). However, sham feeding does not alter transit time during small bowel capsule endoscopy, and chewing gum is not appropriate for patients without masticatory strength (16). Although widely used, early ambulation has not shown any effect on postoperative bowel function (9). Alvimopan has shown promising results in recent years, but its clinical use is still limited due to the potential complications and high cost (5). The current situation prompts both patients and physicians to seek alternative treatment options.

Acupuncture is a non-pharmacological therapy and has been practiced in China for more than 3000 years to treat many types of diseases. Acupuncture involves the stimulation of specific body sites (acupoints) by the insertion or piercing of thin needles through the skin, producing a sensation of “de qi” that has multiple effects on regulating qi and blood circulation (15). These effects then modulate gastrointestinal function, control intestinal inflammation, and suppress acid secretion without adverse effects (17–22). Acupuncture in the ERAS protocol of tumor patients has been shown to be a viable option for restoring gastrointestinal function in a number of surgical procedures, and there is increasing evidence from randomized clinical trials (RCTs) that acupuncture can be used by tumor patients undergoing the ERAS protocol and should be included in the standardized ERAS strategy. Few studies have summarized the effect of acupuncture on gastrointestinal function recovery in tumor patients treated with the ERAS protocol. To provide evidence for decision makers, the main purpose of the present systematic review and meta−analysis was to summarize the evidence and evaluate the effect of acupuncture on gastrointestinal recovery in tumor patients treated with the ERAS strategy.

## 2 Materials and methods

This systematic review and meta-analysis followed the procedures of PROSPERO (https://www.crd.york.ac.uk/prospero/) and a pre-registered review protocol (CRD42023430211), and they were performed according to the Preferred Reporting Items for Systematic Reviews and Meta-Analysis (PRISMA) guidelines. The PRISMA_2020_checklist (23) is included in the **Supplemental Files**. Studies were eligible for inclusion if they reported any of the following outcomes as individual symptoms: time to postoperative oral food intake, time to first flatus, time to first distension and peristaltic sound recovery time (PSRT). The inclusion and exclusion criteria are shown in **Table 1**. A waiver of informed consent was approved by the Institutional Review Board.

**Table 1:** Eligibility criteria related to PICO of inclusion and exclusion criteria.

	Inclusion criteria	Exclusion criteria
P	1.Human studies of any age and sex 2.tumor/cancer patients	Animal studies
I	acupuncture including manual acupuncture or electroacupuncture	1.Non-acupuncture 2.acupuncture combined with medicine 3.either acupressure or laser acupuncture or TEAS without needles 4.auricular acupuncture or fire acupuncture
C	All the patients were treated with an ERAS strategy The control group was sham acupuncture, waitlist control, usual care, or another intervention	1. Patients were treated without an ERAS strategy 2. No control group
O	postoperative symptoms (time to postoperative oral food intake, time to first flatus, time to first distension, PSRT, nausea, vomiting)	preoperative symptoms
S	Peer-reviewed prospective RCTs	Retrospective studies, reviews, meta-analyses, abstracts, letters, congress proceedings, case reports, cross-sectional or case control studies, comments, editorials
L	Articles written in English or Chinese	Articles written in any other language

P, population; I, intervention; C, comparison; O, outcome; S, study design; L, language; TEAS, transcutaneous electrical acupoint stimulation; RCTs, randomized controlled trials; ERAS, enhanced recovery after surgery.

### 2.1 Search strategy

Literature searches were conducted under the supervision of a librarian. The PubMed, Web of Science, EMBASE, Cochrane Library, Wiley Online Library, Scopus, China National Knowledge Infrastructure (CNKI), China Online Journals (COJ), Chinese Biological Medical Database (CBM, SinoMed), Chongqing VIP Chinese Science database and yiigle database were searched from 1997 through June 2023. MeSH terms or title/abstract for PubMed and comparable terms for other databases were used depending on the database, including combinations of “Enhanced Recovery After Surgery”, “Enhanced Postsurgical Recovery”, “Postsurgical Recoveries, Enhanced”, “Postsurgical Recovery, Enhanced”, “Recovery, Enhanced Postsurgical”, “ERAS”, “Fast track surgery”, “Fast-track surgery”, “Accelerated rehabilitation surgery”, “Accelerate recovery surgery”, “Enhanced recovery protocols”, “ERP”, “Enhanced recovery program”, “Fast track”, “ERATS” and “Acupuncture Points”, “Acupuncture Point”, “Point”, Acupuncture”, “Points, Acupuncture”, “Acupoints”, “Acupoint”, “Acupuncture”, “Electro-acupuncture”, “Electroacupuncture”; “Tumor”, “Neoplasm”, “Tumors”, “Neoplasia”, “Neoplasias”, “Cancer”, “Cancers”, “Malignant Neoplasm”, “Malignancy”, “Malignancies”, “Malignant Neoplasms”, “Neoplasm, Malignant”, “Neoplasms”, and “Malignant”. Bibliographies of relevant papers and websites were reviewed, and authors were contacted to obtain complete results. The search was limited to published human studies. The **Supplementary Data** describes the full strategy.

### 2.2 Data extraction and quality assessment

**Figure 1:**
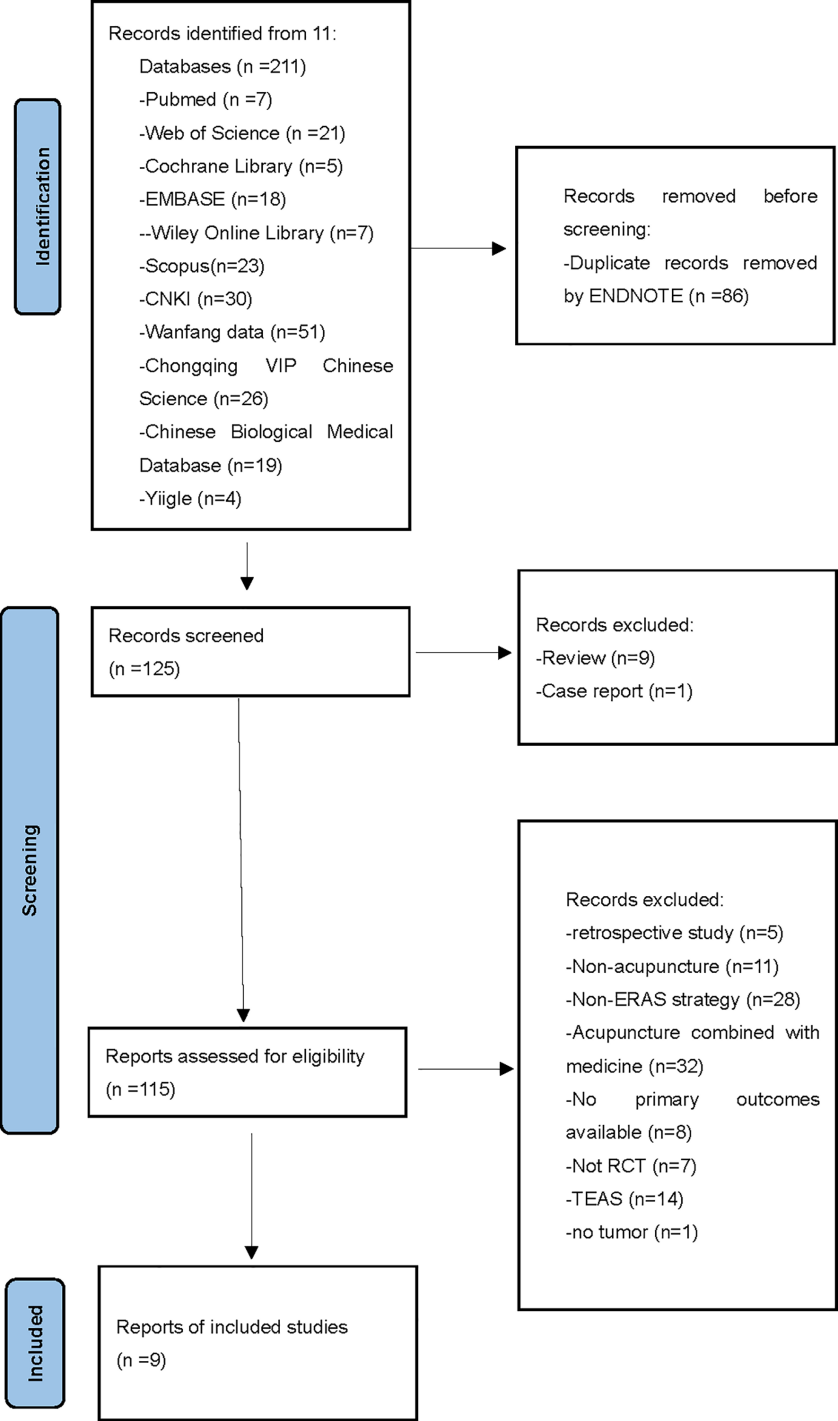
PRISMA flowchart describing the selection process. PRISMA. Preferred Reporting Items for systematic Reviews and Meta-Analysis.

Screening of data was performed using Endnote X8. Studies with duplicate titles were deleted by both Endnote and manual screening. The summary of included studies is shown in **Table 2** and the detailed locations of included acupoints are shown in **Table 3**. Two researchers (J.C. and L.L.) independently reviewed the titles and abstracts using the inclusion and exclusion criteria for initial inclusion. Disagreements between the two researchers were resolved by further review of the full text and discussion with a third researcher (YR.X.). The screening and selection process is detailed in a PRISMA flowchart shown in **Figure 1**.

**Table 2:** Summary of included studies.

Author (year)	Organ/System	Sample Size	Age	Acupoint Stimulation Style	Time Point	Acupoint Stimulation Protocols	Acupoint Stimulation Adverse Reactions	Target outcomes	Jadad Score
Dongxiao Li et al. (2018)	colorectal cancer	82 (42 vs 40)	33-89	acupuncture	6 hours after surgery and twice a day after surgery day until the flatus, defecation and bowel sound recovery	PC6, ST36	none reported	1+2+3+4+5	4
Weijian Zhu et al. (2020)	pancreatic surgery	74 (37 vs 37)	18-78	electroacupuncture	24 hours after surgery, once a day, 6 times in total	ST36, PC6, ST37, ST39, SP4, SP6	none reported	2+3+5	4
Zunxiao Liang et al. (2020)	colorectal cancer	120 (60 vs 60)	36-75	acupuncture	once a day after surgery, 5 times in total	LI10, LI4, ST25, ST36, ST37,	none reported	1+2+3+4	4
Xiaoliang Wu et al. (2020)	gastric cancer	30 (15 vs 15)	?	acupuncture	once after surgery	ST37, ST39	no	1+2+3	4
Weijian Zhu et al. (2021)	colonic surgery	86 (43 vs 43)	18-78	electroacupuncture	24 h after surgery, once a day, 5 times in total	ST36, PC6, ST37, ST39, SP4, SP6	none reported	2+3+5	5
Xueyan Liu et al. (2021)	colorectal surgery	68 (33 vs. 35)	18-80	acupuncture	24 h after surgery, once a day, 4 times in total	ST36, PC6, ST37, LI4	bruise (n=1)	1+2+3+4+5	5
Dan Li et al. (2021)	cervical cancer	100 (50 vs. 50)	18-65	acupuncture	after surgery	SP6, ST36, PC6, ST37, HT7	no reported	2+3+4	4
Kang An et al. (2022)	colorectal surgery	78 (36 vs. 42)	53-80	acupuncture	Before and after surgery,6 times in total	ST36, PC6, ST37, ST25, LI4	no reported	1+2+3	4
Min Guo et al. (2022)	gastrointestinal tumors	64 (32 vs. 32)	40-80	acupuncture	upon the patient's return to the ward, and continued for 7 consecutive days	LI4, PC6, LR3, SP4, ST36, ST37, ST39	no reported	1+2+3	5

peristaltic sound recovery time, PSRT.

1: time to postoperative oral food intake.

2: time to first flatus.

3: time to first defecation.

4: peristaltic sound recovery time, PSRT.

5: pain.

**Table 3:** Description of acupoints.

Acupoint	Detailed location
Zusanli (ST36)	Anterior aspect of the lower leg, about one finger-breadth lateral to the tibia, just inferior to the tibial tuberosity, 3 cuns below Dubi (S 35)
Shangjuxu (ST37)	Anterior aspect of the lower leg, 3 cuns below Zusanli (S 36)
Neiguan (PC6)	at the anterior forearm, 2 cuns above the distal wrist crease, the tendons of palmaris longus, and the flexor carpi radialis muscles
Hegu (LI4)	on the dorsum of the hand, in the middle of the second metacarpal bone on the radial side
Xiajuxu (ST39)	Anterior aspect of the lower leg, 3 cuns below Shangjuxu (ST37)
Gongsun (SP4)	The medial margin of the foot, the anterior lower part of the base of the first metatarsal bone
Sanyinjiao (SP6)	the inner side of the calf, 3 cuns above the ankle tip of the foot, behind the medial edge of the tibia
Tianshu (ST25)	in the abdomen, horizontally flat in the navel, 2 cuns apart from the anterior midline
Shenmen (HT7)	at the wrist, at the ulnar end of the transverse striation on the palmar side of the wrist, at the radial depression of the flexor carpi ulnaris tendon
Shousanli (LI10)	On the radial side of the back of the forearm, 2 cuns below the elbow line
Taichong (LR3)	at the dorsum of the foot, in the depression distal to the junction of the first and second metatarsal bones

1 cun ≈ 3.33 cm.

Two researchers (J.C. and L.L.) evaluated the quality of included studies using a modified Jadad Scale. The following items were evaluated: 1) random sequence generation; 2) allocation concealment; 3) blinding of participants and personnel or outcome assessment; and 4) reporting of the number of dropouts and reasons for withdrawal. Each study was scored on a scale of 0–7 with 0–3, 4–5, and 6–7 indicating low quality, moderate quality, and high quality, respectively (24). Two researchers (J.C. and L.L.) independently extracted the data and then assessed the quality of each article, evaluating the risk of bias (RoB) using the Cochrane Collaboration criteria. The Cochrane tool is comprised of the following seven domains: 1) random sequence generation; 2) allocation concealment; 3) blinding of participants and personnel; 4) blinding of outcome assessment; 5) incomplete outcome data; 6) selective reporting; and 7) other bias. Disagreements were resolved through discussion or by involving a third author (YR.X.).

For each article, the following information was extracted: authors; country where the study was performed; gastrointestinal symptoms and other symptoms; surgical organs or systems; study design; sample size; treatment period; acupuncture protocols; acupuncture style; adverse effect; and significant results between the acupuncture and control groups. Discrepancies identified in the study selection and data extraction were discussed with another researcher to reach an agreement.

### 2.3 Meta-analysis and synthesis of evidence

The meta-analysis was performed using software of Rev-Man 5.4.1, Stata/MP 17.0 and R v4.3.0. The acupuncture group was compared to the control group for each item. The standardized mean difference (SMD) and corresponding 95% confidence interval (CI) were measured for the effect sizes of continuous outcomes. The risk ratio (RR) and corresponding 95% CI were measured for the effect sizes of binary outcomes. Heterogeneity was quantified using I^2^ statistics. Fixed-effects methods were used when heterogeneity was low (I^2^ ≤ 50%), and random-effects methods were used when heterogeneity was high (I^2^>50%). Only studies with fully available data were included in the analysis, and the authors of the original studies were contacted if the data were not complete. Analysis was continued if authors had not responded for a period of more than 4 weeks. Sensitivity analysis was conducted by comparing the effect sizes of the remaining RCTs after removing each included RCT one at a time. Additionally, the authors conducted a meta-regression analysis to explore whether the types of acupuncture (manual acupuncture or electroacupuncture), samples and the surgical organs were associated with efficacy, if so, a subgroup analysis was performed. An overall p-value was determined and considered statistically significant when p<0.05.

## 3 Results

The search of the databases yielded 211 journal articles. After excluding 86 duplicates, 125 articles remained for further screening. Nine RCTs that examined one of the gastrointestinal functions of interest were evaluated for RoB and considered for the meta-analysis.

### 3.1 Characteristics of the studies and quality of evidence

Among the nine studies (702 patients) reviewed, the most commonly recorded postoperative gastrointestinal functional events were time to first flatus ( (25–33) (n=9) and time to first distension (25–33) (n=9) followed by time to postoperative oral food intake (25, 26, 29, 31–33) (n=6) and PSRT (26, 30, 32) (n=3). Pain was assessed in four of the nine studies, and four of which were eligible for further analysis. All of the nine studies were conducted in China, with eight studies were in Chinese and one study written in English (33). Seven studies used manual acupuncture (25, 26, 29–33), and two studies used electroacupuncture (27, 28). The sample size of the studies ranged from 30 to 120. All acupuncture treatments were performed during the peri-operative period. The study design and type of control groups in the nine studies were acupuncture versus usual treatment, and only one study compared acupuncture versus sham treatment (33). With the exception of one study on cervical carcinoma surgery (30), the other included studies encompassed surgery for gastrointestinal tumors. Although different acupoints were used in the various studies, the same acupuncture protocol was used for a specific type of ERAS strategy. The most commonly used acupoints were Zusanli (ST36) (n=8), Shangjuxu (ST37) (n=8), and Neiguan (PC6) (n=7) followed by Hegu (LI4) (n=4), Xiajuxu (ST39) (n=4), Sanyinjiao (SP6) (n=3), Gongsun (SP4) (n=3), Tianshu (ST25) (n=2), Shenmen (HT7) (n=1), Shousanli (LI10) (n=1) and Taichong (LR3) (n=1). Most of the acupoints were located on the limbs, and both ST36 and ST37 were located on the Foot Yangming stomach meridian.

**Figure 2:**
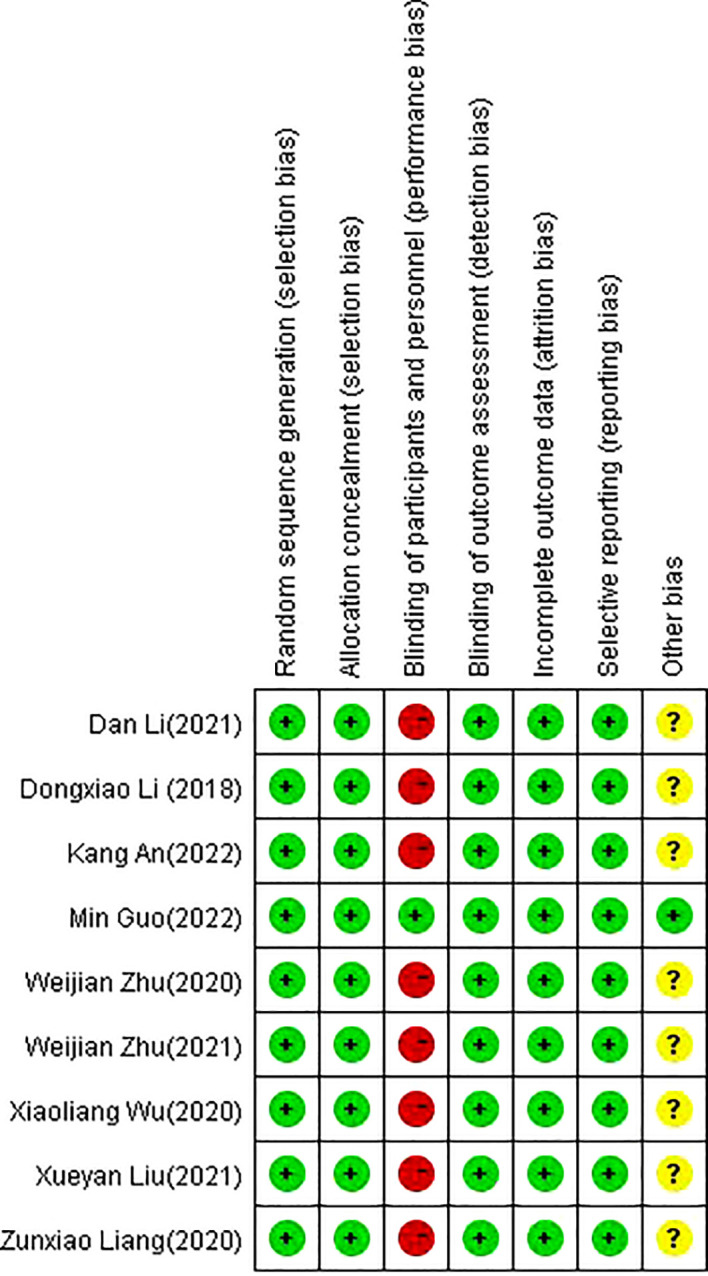
The summary of the risk of bias.

**Figure 3:**
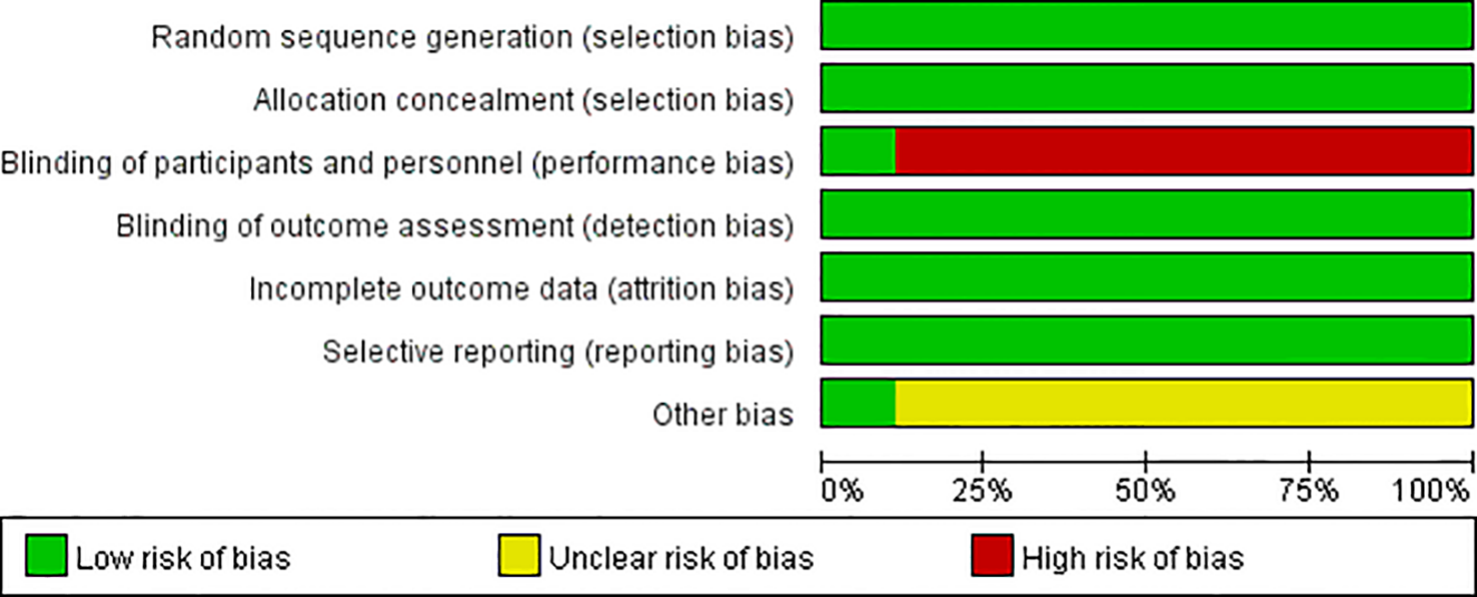
The risk of bias graph.

For the nine RCTs included in the present systematic review and meta−analysis, the RoB values were identified. Although the acupuncturist was not blinded in any of these studies, with the exception of one study that was considered questionable due to some incomplete cases (26), all other included studies were rated as low risk according to the RoB rating. **Figures 2**, **3** show summaries of the included RCTs and RoB values, respectively.

### 3.2 Adverse events

Adverse events related to acupuncture were observed in two studies, and bruising was reported in only one case (26). In seven studies, the adverse events were not mentioned or reported. Based on the long history and available data, acupuncture is generally considered a safe treatment with only mild adverse events (34).

### 3.3 Meta-analysis

**Figure 4:**
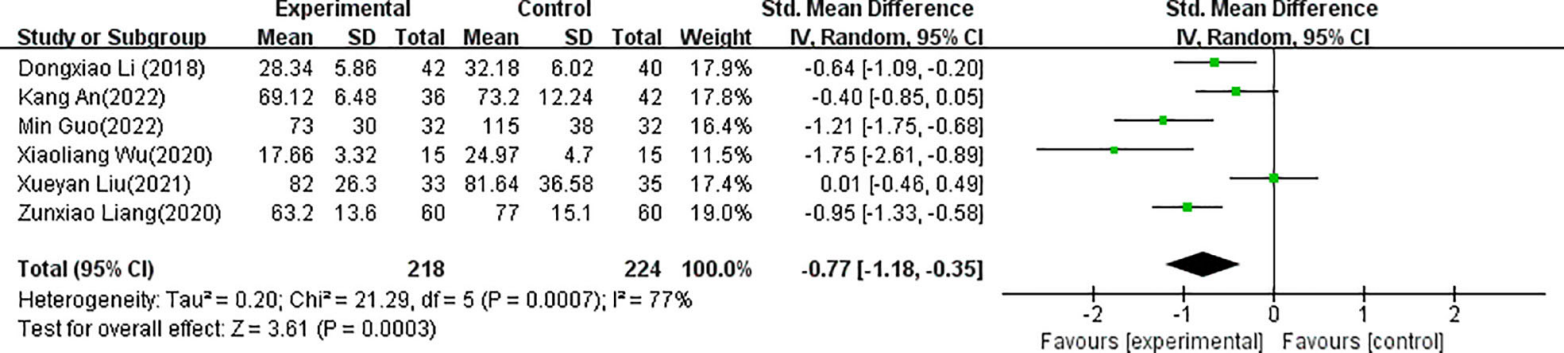
Forest plots of the comparison of acupuncture with control groups for time to postoperative oral food intake.

**Figure 5:**
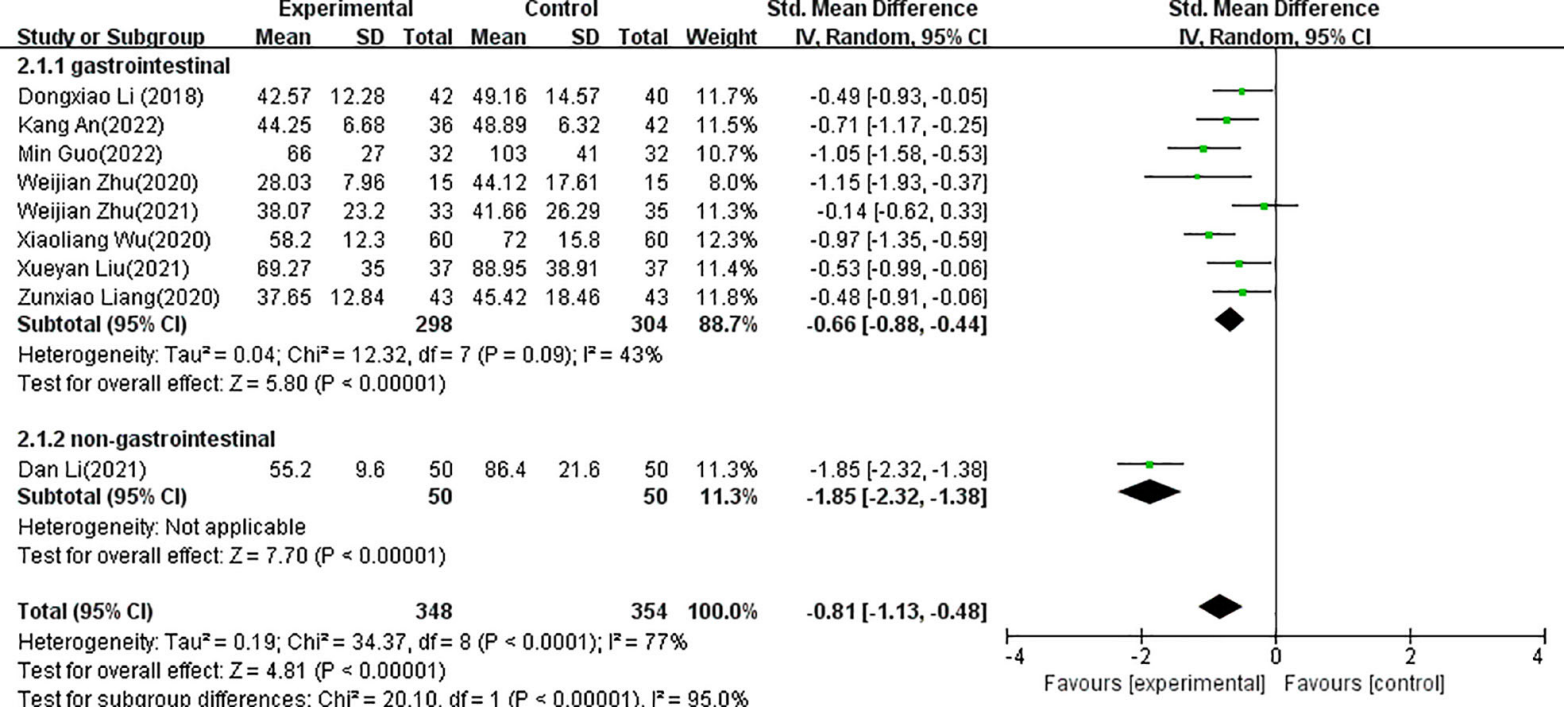
Forest plots of the comparison of acupuncture with control groups for postoperative time to first flatus.

**Figure 6:**
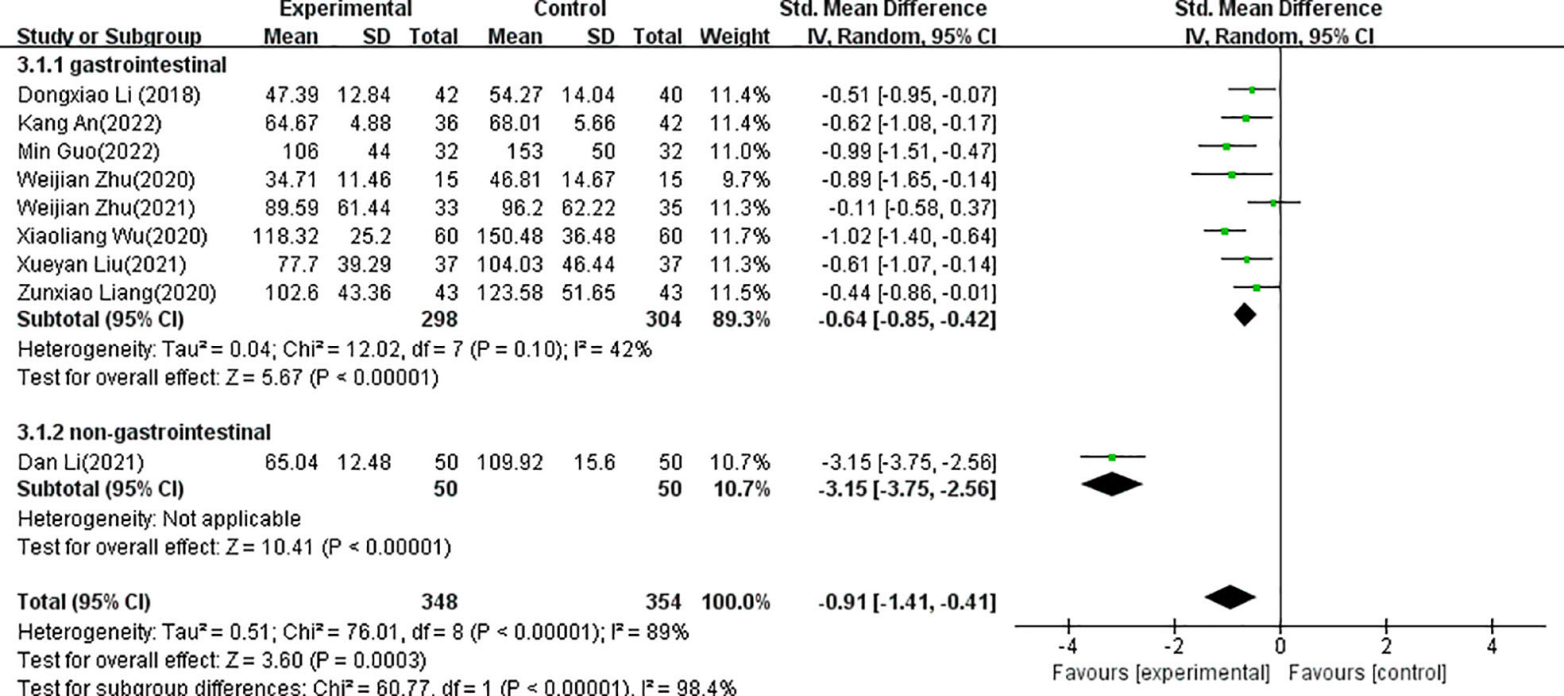
Forest plots of the comparison of acupuncture with control groups for postoperative time to first defecation.

**Figure 7:**
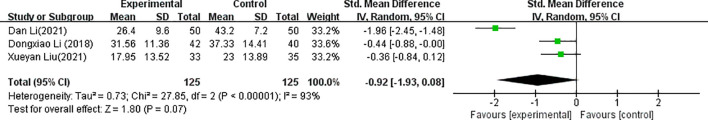
Forest plots of the comparison of acupuncture with control groups for postoperative peristaltic sound recovery time.

**Figure 8:**
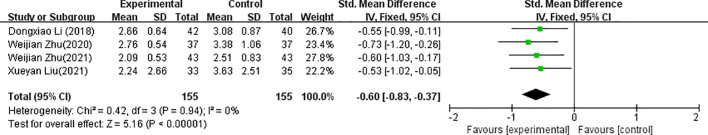
Forest plots of the comparison of acupuncture with control groups for postoperative pain.

The forest plots for acupuncture versus control groups for each symptom are shown in **Figures 4**–**8**. There was a statistically significant positive effect of acupuncture on reducing time to postoperative oral food intake [standardized mean difference (SMD) = -0.77, 95% confidence interval (CI) -1.18 to -0.35], time to first flatus (SMD=-0.81, 95% CI -1.13 to -0.48), time to first defecation (SMD=-0.91, 95% CI -1.41 to -0.41, PSRT (SMD=-0.92, 95% CI -1.93 to 0.08), and pain intensity (SMD=-0.60, 95% CI -0.83 to -0.37) with statistical heterogeneity of 77%, 77%, 89%, 93%, and 0%, respectively. A sensitivity analysis of the meta-analysis was conducted by excluding included trials one by one. The results showed that all the outcomes showed no obvious modification of the overall effect. With the high heterogeneities of defecation and flatus, meta-regression according to the types of acupuncture (manual acupuncture or electroacupuncture), samples and the surgical organs have been done with the software of Stata, the results showed that types of acupuncture (manual acupuncture or electroacupuncture) and samples did not influence the overall heterogeneity (P>0.05). The different of surgical organs influence the overall heterogeneity obviously (P<0.05). A subgroup analysis of defecation and flatus was performed according to the surgical organs. The results showed that there was a statistically significant positive effect of acupuncture on time to first flatus (SMD=-0.66, 95% CI -0.88 to -0.44) and time to first defecation (SMD=-0.64, 95% CI -0.85 to -0.42 with statistical heterogeneity of 43%, and 42%, respectively in gastrointestinal tumors.

### 3.4 Strengths and limitations

The main strength of the present study was the extensive search of Chinese literature as acupuncture was developed in China and is mainly used in China. The limitations of the present systematic review and meta−analysis comprised the exclusive inclusion of studies published in English and Chinese. All included studies were conducted in China, and the patients were all Chinese, thus limiting the scope of the study because there may be other acupuncture RCTs published in Korean, Japanese, or other languages. Future systematic reviews and meta−analyses should include additional studies in more languages and more countries. In addition, the present study investigated only gastrointestinal function and pain control, while other symptoms were not considered. Future studies should measure multiple co-occurring symptoms using an exhaustive scale in different ERAS strategies. When sufficient studies have been published, more efficient and appropriate methods for analysis will be available. Finally, the present systematic review and meta−analysis included only pre-discharge items, and no single study reported long-term or follow-up symptoms, which should be included in future analyses.

## 4 Discussion

The present systematic review and meta−analysis evaluated acupuncture (manual acupuncture and electroacupuncture) in restoring gastrointestinal function in tumor patients treated with an ERAS protocol.

ERAS is a surgical strategy introduced in 1997 by a group of general surgeons from Northern Europe led by Henrik Kehlet to promote recovery after surgery (35). This strategy was later expanded by the ERAS Study Group, resulting in structured and evidence-based perioperative protocols, as published by the ERAS Society and other groups, such as the Society of American Gastrointestinal and Endoscopic Surgeons, to replace traditional surgical strategies (2). Gradual recognition of the improvements in postoperative outcomes has resulted in the ERAS strategy now being standard of care for most surgical procedures of tumor patients (36–38).

The surgical stress and triggered neuroendocrine responses are the main reasons for impeding postoperative recovery of tumor patients, with the gastrointestinal tract being the main target organ of the neuroendocrine system (2, 39). Stress-related mucosal disease is an important manifestation of gastrointestinal dysfunction after surgery, ranging from ileus to less common complications, such as gastrointestinal bleeding and mesenteric ischemia, both of which are associated with high mortality (40). Although prophylaxis was used long before the ERAS strategy, the advent of the ERAS strategy has increased the importance of prophylaxis in perioperative care and focused attention on the true nature of postoperative gastrointestinal dysfunction. However, despite the continuous improvement in treatment, truly effective interventions for gastrointestinal dysfunction are still limited (39, 41). As pain control is considered an integral part of the ERAS protocol and opioid therapy remains the cornerstone of postsurgical pain control, opioids play an important part in the hindering of recovery due to their well-recognized gastrointestinal side effects (10).

As a type of non-pharmacological therapy with a long history in China, acupuncture has been regarded as an effective treatment for diseases of the gastrointestinal tract for thousands of years (42). With research into its mechanism and efficacy, acupuncture has been recognized worldwide, and patients’ expectations of acupuncture have increased in recent years (43). To date, manual acupuncture and electroacupuncture are the two most common types of acupuncture in the world. Manual acupuncture, which is traditional acupuncture, involves stimulating acupuncture points located on specific parts of the body by inserting fine metal (formerly silver but currently stainless steel) needles and then manipulating them (44). In recent years, electroacupuncture has been developed based on manual acupuncture, in which a weak electric current is connected to the needle and it is thought to enhance the effect of manual acupuncture (45). However, whether electroacupuncture is more effective than manual acupuncture in continuously stimulating acupoints is controversial. Previous studies have shown that manual acupuncture, but not electroacupuncture, has beneficial effects on associated disability, strength, and pain in musculoskeletal epicondylalgia in the short term (46).

The exact biological mechanisms of the effects of acupuncture on gastrointestinal tract functions are still being researched. In general, it is considered that the somatic afferents present in the acupoints sequentially transmit sensory signals to the spinal cord, brainstem and hypothalamic neurons after stimulating the acupuncture points. The integration of the information in the brain stimulates other multiple signaling pathways, including neuroendocrine pathways, neuroimmune pathways, and others, which eventually influence the functional activities of the gastrointestinal tract through the nervous, endocrine, and digestive systems (17, 19, 47–50). Acupuncture has not yet been included in the global ERAS strategy guidelines. Some previous studies have experimentally tested acupuncture and demonstrated its effectiveness for restoring gastrointestinal function in tumor patients receiving the ERAS strategy. In the present systematic review and meta−analysis, we summarized the evidence and evaluated the effect of acupuncture on gastrointestinal recovery in tumor patients subjected to the ERAS strategy, providing evidence for its inclusion in the ERAS strategy. Nine studies (702 patients) were included in the present systematic review and meta−analysis. Time to first flatus and time to first distension were the most commonly used indices to assess recovery of gastrointestinal function. The time to postoperative oral food intake and PSRT were also investigated as indices of recovery of gastrointestinal function after surgery. The present meta-analysis indicated that acupuncture significantly promoted the recovery of gastrointestinal function compared to the control treatment. In addition, acupuncture showed significant effects in reducing time to postoperative oral food intake, time to first flatus, time to first distension, and PSRT compared to the control treatment.

The ERAS strategy is currently applied in most tumor surgical specialties. However, the included studies mainly investigated surgery for gastrointestinal tumors (n=8). For certain types of disease, the included studies used a standardized acupuncture research protocol, and ST36 and ST37 were the most commonly used acupoints. ST36 and ST37 have been widely used in clinical practice to promote gastrointestinal function. Compared to drugs, acupuncture stimulation of ST36 and ST37 shows better results in improving diarrhea predominant in irritable bowel syndrome (51) and in treating postoperative ileus (52). Both ST36 and ST37 belong to the meridian called the Foot Yangming stomach meridian, which is responsible for the entire digestive system and not just for the anatomical stomach. The mechanisms may involve the regulation of multiple systems. Stimulation of ST36, ST37, and other acupoints in the limbs called “de qi” in Traditional Chinese Medicine elicits transmission of multiple nervous system signals that help reduce autonomic, neuroendocrine, and immunological responses caused by surgical stress. In addition to the common therapeutic effect and mechanism of acupoints, ST36 has been reported to put the second somatosensory cortex in the “de qi” state, with various structures of the nervous system showing consistent attenuation signals, including the para-structure and marginal structure of the telencephalon, brainstem, interbrain, subcortical area, and cerebellar cortex (53, 54). Needling at ST37 significantly inhibits the discharge of locus coeruleus neurons triggered by colon distension (55). The combined stimulation of ST36 and ST37 exhibits a dual regulatory function by balancing different signaling pathways, such as the Toll-like receptor 4 (TLR4)/nuclear factor kappa B (NF-kB) and mitogen-activated protein kinase (MAPK) signaling pathways as well as the cholinergic anti-inflammatory pathway (56, 57). PC6 regulates the function of the stomach qi and subsequently prevents nausea and vomiting (58), and it has been widely used in the prevention and treatment of postoperative gastrointestinal dysfunction (59). Previous studies have found that electroacupuncture at PC6 reduces the inhibition of efferent vagal motor fibers, primarily by inhibiting gamma-aminobutyric acid (GABA) transmission to dorsal motor nucleus of the vagus, thereby promoting efferent vagus nerve activity and increasing gastric motility (60).

As it is difficult to blind acupuncturists, the quality of control groups is challenging. In general, there are two types of sham acupuncture used in research. The first type uses acupuncture needles that penetrate the skin at non-acupuncture points and shallowly insert into acupuncture points. One study in the present systematic review and meta−analysis used needles that were inserted at a depth of 10 mm at nearby non-acupuncture points and were inserted at a depth of 2 mm in the sham acupuncture group (33), whereas the usual depth of acupuncture in the clinic is 25 mm. However, this type of sham acupuncture control has been criticized because it may have a therapeutic effect; the point in close proximity is usually another acupuncture point, and superficial penetration or insertion may also cause stimulation. Another type of sham acupuncture is a type of device with a non-penetrating placebo needle, such as Streitberger, Park, or Takakura needles, but no study in the present meta-analysis used these non-penetrating placebo needles. Compared to Streitberger or Park needles, the Takakura double-blind needle, which consists of a placebo skin-touch needle and a penetration needle, is considered blind by both participants and acupuncturists because neither the participant nor the acupuncturist can determine by feel or sight which needle (placebo or penetration) is being used. In addition to cost factors and access limitations, the validity of the Takakura needle needs to be further investigated as it is difficult to blind experienced acupuncturists. However, compared to other sham acupuncture devices, this device is currently the best option for blinding and should be considered for double-blind acupuncture RCTs in the future.

The use of multimodal analgesia for optimal postoperative pain management is one of the most important components of the ERAS strategy (10). Optimal pain relief is also critical for patients to reduce surgical stress and participate in early mobilization after any surgery (61). Although opioids are still widely used in surgery (10), the influence of opioids, such as morphine, on cancer progression is controversial (62). In the context of the opioid crisis and the highly restrictive use of opioids in the ERAS strategy, the risk of undertreatment of acute pain and the development of persistent chronic pain after surgery is rising. Moreover, there is an increasing focus on minimizing the non-narcotic use of the ERAS strategy. In addition, little research has been conducted on the efficacy of pain management after discharge (36). Because pain control is considered an integral part of the ERAS strategy and drug treatment is flawed to some extent (9), acupuncture, a non-pharmacological therapy, is a good choice. The analgesic effect of acupuncture has been widely demonstrated, and this effect may even be sustained over a longer period of time (63). In the present meta-analysis, four of the nine studies (two manual acupuncture studies and two electroacupuncture studies) evaluated both the effect of acupuncture on pain control and on the recovery of gastrointestinal function. The gastrointestinal function recovery and pain control are currently recognized as two of the three fundamental aspects of recovery from surgery along with mobilization (64). The present review and meta-analysis demonstrated that acupuncture has an effect on pain control while improving gastrointestinal function.

Acupuncture has also been found to be an effective and simple treatment method for restoring gastrointestinal function in the elderly and young children (65, 66). Acupuncture has been found to be useful for improved recovery of gastrointestinal function, and pain control in the elderly.

## 5 Conclusions

Acupuncture significantly promotes recovery of gastrointestinal function and pain control in tumor patients treated with the ERAS strategy, with ST36 and ST37 being the most commonly used acupuncture points. Although the safety of acupuncture was poorly reported in the included studies, acupuncture is recommended to be included in the ERAS strategy for tumor patients seeking nonpharmacologic treatment for recovery of gastrointestinal function and pain control. Due to the problems associated with blinding in all included RCTs, more rigorous studies are needed to confirm the efficacy of acupuncture in promoting recovery of gastrointestinal function and pain control in tumor patients treated with the ERAS strategy. Both high-quality and large-scale randomized clinical trials are needed in the future to verify these results and develop a standardized strategy for acupuncture in the ERAS protocol. The present results will hopefully lead to improved recovery of surgical patients with cancers.

## Data availability statement

The original contributions presented in the study are included in the article/**Supplementary Material**. Further inquiries can be directed to the corresponding author.

## Author contributions

JC and YX were responsible for acquisition, interpretation, and drafting the article. LL substantially contributed to the data analysis. YL and TF critically revised the work for important intellectual content. YL was included in the article drafting and also critically revised the work. All authors provided final approval of the version to be published and agree to be accountable for all aspects of the work.
