# Supplementary material for: Effect of acupuncture inclusion in the enhanced recovery after surgery protocol on tumor patient gastrointestinal function: a systematic review and meta-analysis of randomized controlled studies
Source: Front Oncol. 2023 Aug 15;13:1232754. doi: 10.3389/fonc.2023.1232754 (PMC10465796; doi:10.3389/fonc.2023.1232754)
Supplement: Supplementary file 1 [file DataSheet_1.docx]

1. **Pubmed**

**#1: "Enhanced Recovery After Surgery"[MeSH Terms]**

**#2: "Enhanced Postsurgical Recovery"[Title/Abstract] OR "ERAS"[Title/Abstract] OR "Fast-track surgery"[Title/Abstract] OR "Fast-track surgery"[Title/Abstract] OR "Accelerated rehabilitation surgery"[Title/Abstract] OR "Enhanced recovery protocols"[Title/Abstract] OR "ERP"[Title/Abstract] OR "enhanced recovery program"[Title/Abstract] OR "fast track"[Title/Abstract] OR "ERATS"[Title/Abstract]**

**#3:(#1) OR (#2)**

**#4: Neoplasms[MeSH Terms]**

**#5: "Tumor"[Title/Abstract] OR "Neoplasm"[Title/Abstract] OR "Tumors"[Title/Abstract] OR "Neoplasia"[Title/Abstract] OR "Neoplasias"[Title/Abstract] OR "Cancer"[Title/Abstract] OR "Cancers"[Title/Abstract] OR "Malignant Neoplasm"[Title/Abstract] OR "Malignancy"[Title/Abstract] OR "Malignancies"[Title/Abstract] OR "Malignant Neoplasms"[Title/Abstract] OR "neoplasm malignant"[Title/Abstract] OR "neoplasms malignant"[Title/Abstract]#6: Neoplasms[MeSH Terms]**

**#6: #4 OR #5**

**#7:"Acupuncture Points"[All Fields] OR "Acupuncture Point"[All Fields] OR "point acupuncture"[All Fields] OR "points acupuncture"[All Fields] OR ("acupoint s"[All Fields] OR "Acupuncture Points"[MeSH Terms] OR ("acupuncture"[All Fields] AND "points"[All Fields]) OR "Acupuncture Points"[All Fields] OR "acupoint"[All Fields] OR "acupoints"[All Fields]) OR ("acupoint s"[All Fields] OR "Acupuncture Points"[MeSH Terms] OR ("acupuncture"[All Fields] AND "points"[All Fields]) OR "Acupuncture Points"[All Fields] OR "acupoint"[All Fields] OR "acupoints"[All Fields]) OR ("acupunctural"[All Fields] OR "acupuncture"[MeSH Terms] OR "acupuncture"[All Fields] OR "acupuncture therapy"[MeSH Terms] OR ("acupuncture"[All Fields] AND "therapy"[All Fields]) OR "acupuncture therapy"[All Fields] OR "acupuncture s"[All Fields] OR "acupunctured"[All Fields] OR "acupunctures"[All Fields] OR "acupuncturing"[All Fields]) OR "electro-acupuncture"[All Fields] OR ("electroacupuncture"[MeSH Terms] OR "electroacupuncture"[All Fields] OR "electroacupuncturing"[All Fields])**

**#8: (#3) AND (#6) AND (#/7)**

**Filters: Clinical Trial, Randomized Controlled Trial, from 1997/1/1 - 2023/06/30 Altogether,7 articles were included.**

1. **Web of Science**

**#1: TS=(Enhanced Recovery After Surgery) OR TS=(Enhanced Postsurgical Recovery) OR TS=(Postsurgical Recoveries, Enhanced) OR TS=(Postsurgical Recovery, Enhanced)OR TS=(Recovery, Enhanced Postsurgical) OR TS=(ERAS) OR TS=(fast track surgery) OR TS=(Fast-track surgery) OR TS=(Accelerated rehabilitation surgery) OR TS=(Accelerate recovery surgery) OR TS=(Enhanced recovery protocols) OR TS=(ERP) OR TS=(enhanced recovery program) OR TS=(fast track ) OR TS=(ERATS)**

**#2: TS= (Acupuncture Points) OR TS= (Acupuncture Point) OR TS= (Point, Acupuncture) OR TS= (Points, Acupuncture) OR TS=(Acupoints) OR TS=(Acupoint) OR TS=(Acupuncture) OR TS=(electro-acupuncture) OR TS=(electroacupuncture)**

**#3: TS= (“Neoplasms” OR “Tumor” OR “Neoplasm” OR “Tumors” OR “Neoplasia” OR “Neoplasias” OR “Cancer” OR “Cancers” OR “Malignant Neoplasm” OR “Malignancy” OR “Malignancies” OR “Malignant Neoplasms” OR “Neoplasm, Malignant” OR “Neoplasms, Malignant”)**

**#4: #1 AND #2 AND #3** **and clinical trial (publication type)**

**Altogether,21 articles were included.**

**（3）EMBASE**

**#1: 'enhanced recovery after surgery'/exp**

**#2: 'enhanced recovery after surgery'/exp OR 'enhanced recovery after surgery' OR (enhanced AND ('recovery' OR 'recovery'/exp OR recovery) AND after AND ('surgery' OR 'surgery'/exp OR surgery)) OR 'enhanced postsurgical recovery':ti,ab,kw OR 'postsurgical recoveries, enhanced':ti,ab,kw OR 'postsurgical recovery, enhanced':ti,ab,kw OR 'recovery, enhanced postsurgical':ti,ab,kw OR eras:ti,ab,kw OR 'fast track surgery':ti,ab,kw OR 'fast-track surgery':ti,ab,kw OR 'accelerate recovery surgery':ti,ab,kw OR 'enhanced recovery protocols':ti,ab,kw OR erp:ti,ab,kw OR 'enhanced recovery program':ti,ab,kw OR 'fast track':ti,ab,kw OR erats:ti,ab,kw OR 'accelerated rehabilitation surgery':ti,ab,kw**

**#3: 'acupuncture point'/exp**

**#4: 'acupuncture'/exp**

**#5:'acupuncture points'/exp OR 'acupuncture points' OR (('acupuncture' OR 'acupuncture'/exp OR acupuncture) AND points) OR 'acupuncture point':ti,ab,kw OR 'point, acupuncture':ti,ab,kw OR 'points, acupuncture':ti,ab,kw OR acupoints:ti,ab,kw OR acupoint:ti,ab,kw OR acupuncture:ti,ab,kw OR 'electro acupuncture':ti,ab,kw OR electroacupuncture:ti,ab,kw**

**#6:'neoplasm'/exp**

**#7'tumor'/exp OR 'tumor' OR 'neoplasm'/exp OR 'neoplasm' OR 'tumors'/exp OR 'tumors' OR 'neoplasia'/exp OR 'neoplasia' OR 'neoplasias' OR 'cancer'/exp OR 'cancer' OR 'cancers'/exp OR 'cancers' OR 'malignant neoplasm'/exp OR 'malignant neoplasm' OR 'malignancy'/exp OR 'malignancy' OR 'malignancies'/exp OR 'malignancies' OR 'malignant neoplasms' OR 'neoplasm, malignant' OR 'neoplasms, malignant':**

**#8: #1 OR #2**

**#9: #3 OR #4 OR #5**

**#10: #6 OR #7**

**#11:#8 AND #9 AND #10**

**#12: #11 AND 'article'/it**

**Altogether,18 articles were included.**

**(4) Cochrane Library**

**#1: MeSH descriptor: [Enhanced Recovery After Surgery] explode all trees**

**#2:Enhanced Recovery After Surgery in Title Abstract Keyword OR Enhanced Postsurgical Recovery in Title Abstract Keyword OR Postsurgical Recoveries, Enhanced in Title Abstract Keyword OR Postsurgical Recovery, Enhanced in Title Abstract Keyword OR Recovery, Enhanced Postsurgical in Title Abstract Keyword OR ERAS in Title Abstract Keyword OR fast track surgery in Title Abstract Keyword OR Fast-track surgery in Title Abstract Keyword OR Accelerated rehabilitation surgery in Title Abstract Keyword OR Accelerate recovery surgery in Title Abstract Keyword OR Enhanced recovery protocols in Title Abstract Keyword OR ERP in Title Abstract Keyword OR enhanced recovery program in Title Abstract Keyword OR fast track in Title Abstract Keyword OR ERATS**

**#3: #1 or #2**

**#4: MeSH descriptor: [Neoplasms] explode all trees**

**#5: (“Tumor” OR “Neoplasm” OR “Tumors” OR “Neoplasia” OR “Neoplasias” OR “Cancer” OR “Cancers” OR “Malignant Neoplasm” OR “Malignancy” OR “Malignancies” OR “Malignant Neoplasms” OR “Neoplasm, Malignant” OR “Neoplasms, Malignant”):ti,ab,kw**

**#6: #4 or #5**

**#7: MeSH descriptor: [Acupuncture Points] explode all trees**

**#8: MeSH descriptor: [Acupuncture] explode all trees**

**#9: Acupuncture in All Text OR electro-acupuncture in All Text OR electroacupuncture in All Text OR acupoint in All Text OR Acupoints in All Text**

**#10: #7 or #8 or #9**

**#3 and #6 and #10**

**Altogether,5 article was included.**

(5) **Wiley Online Library**

**7 results for"Enhanced Recovery After Surgery" in Abstract and "acupoint OR acupuncture OR acupoints OR electroacupuncture " anywhere and "Tumor OR Neoplasm OR Neoplasms OR Tumors OR Neoplasia OR Neoplasias OR Cancer OR Cancers OR “Malignant Neoplasm” OR Malignancy OR Malignancies OR “Malignant Neoplasms” OR “Neoplasm, Malignant” OR “Neoplasms, Malignant”" in Abstract**

**(6) Scopus**

**( TITLE-ABS-KEY ( acupoint OR acupuncture OR acupoints OR electroacupuncture ) AND TITLE-ABS-KEY ( "Tumor" OR "Neoplasm" OR "Tumors" OR "Neoplasia" OR "Neoplasias" OR "Cancer" OR "Cancers" OR "Malignant Neoplasm" OR "Malignancy" OR "Malignancies" OR "Malignant Neoplasms" OR "Neoplasm, Malignant" OR "Neoplasms, Malignant" ) AND TITLE-ABS-KEY ( "Enhanced Recovery After Surgery" OR “Enhanced Postsurgical Recovery" OR "Postsurgical Recoveries, Enhanced" OR "Postsurgical Recovery, Enhanced" OR "Recovery, Enhanced Postsurgical" OR ERAS OR "fast track surgery" OR "Fast-track surgery" OR "Accelerated rehabilitation surgery" OR "Accelerate recovery surgery" OR "Enhanced recovery protocols" OR ERP OR "enhanced recovery program" OR "fast track")**

**And article**

**Altogether,23 article was included.**

**(7) CNKI**

**Topic: “acupoint or acupuncture or acupoints or electroacupuncture” and ERAS and “ “Tumor” OR “Neoplasm” OR “Tumors” OR “Neoplasia” OR “Neoplasias” OR “Cancer” OR “Cancers” OR “Malignant Neoplasm” OR “Malignancy” OR “Malignancies” OR “Malignant Neoplasms” OR “Neoplasm, Malignant” OR “Neoplasms, Malignant””**

**In Chinese：**

**针灸 + 体针 + 穴位 + 刺灸 + 针刺 + 电针 + 针**

**加速康复外科 + 加速康复 + 术后加速康复 + 加速术后康复 + 快速康复 + 术后快速康复 + 快速术后康复**

**癌症 + 肿瘤 + 癌**

**30 results (21 in Chinese and 9 in English)**

**(8) China Online Journals (COJ)**

**abstract:“acupoint or acupuncture or acupoints or electroacupuncture” and ERAS and “ “Tumor” OR “Neoplasm” OR “Tumors” OR “Neoplasia” OR “Neoplasias” OR “Cancer” OR “Cancers” OR “Malignant Neoplasm” OR “Malignancy” OR “Malignancies” OR “Malignant Neoplasms” OR “Neoplasm, Malignant” OR “Neoplasms, Malignant””**

**In Chinese：**

**(题名或关键词:(针灸 OR 体针 OR 穴位 OR 刺灸 OR 针刺 OR 电针 OR 针) and 题名或关键词:(加速康复外科 OR加速康复 OR 术后加速康复 OR 加速术后康复 OR 快速康复 OR 术后快速康复 OR 快速术后康复) and 题名或关键词:( 癌症 OR 肿瘤 OR 癌)) and Date:1997-***

**51 results**

**(9) Chongqing VIP Chinese Science**

**abstract: “acupoint or acupuncture or acupoints or electroacupuncture” and ERAS and “ “Tumor” OR “Neoplasm” OR “Tumors” OR “Neoplasia” OR “Neoplasias” OR “Cancer” OR “Cancers” OR “Malignant Neoplasm” OR “Malignancy” OR “Malignancies” OR “Malignant Neoplasms” OR “Neoplasm, Malignant” OR “Neoplasms, Malignant””**

**In Chinese：**

**abstract: “加速康复 OR 快速康复” and “穴位 OR 针灸 OR 体针 OR 针刺 OR 电针OR 针”and “癌症 OR 肿瘤 OR 癌” and publication year from 1997 to 2023**

**26 results**

**(10)** **Chinese Biological Medical Database (CBM, SinoMed)**

**abstract: “acupoint or acupuncture or acupoints or electroacupuncture” and ERAS and “ “Tumor” OR “Neoplasm” OR “Tumors” OR “Neoplasia” OR “Neoplasias” OR “Cancer” OR “Cancers” OR “Malignant Neoplasm” OR “Malignancy” OR “Malignancies” OR “Malignant Neoplasms” OR “Neoplasm, Malignant” OR “Neoplasms, Malignant””**

**In Chinese：**

**[(( "癌症"[标题:智能] OR "肿瘤"[标题:智能] OR "癌"[标题:智能]) AND( "加速康复"[标题:智能] OR "术后加速康复"[标题:智能] OR "加速术后康复"[标题:智能] OR "快速康复"[标题:智能] OR "术后快速康复"[标题:智能] OR "快速术后康复"[标题:智能]) AND( "针灸"[标题:智能] OR "体针"[标题:智能] OR "穴位"[标题:智能] OR "刺灸"[标题:智能] OR "针刺"[标题:智能] OR "电针"[标题:智能] OR "针"[标题:智能])) AND ("随机对照试验"[文献类型])](javascript:toDoRelimitSearch();)**

**19 results**

**(11) Yiigle (中华医学期刊全文数据库)**

**Topic: “acupoint or acupuncture or acupoints or electroacupuncture” and ERAS and “ “Tumor” OR “Neoplasm” OR “Tumors” OR “Neoplasia” OR “Neoplasias” OR “Cancer” OR “Cancers” OR “Malignant Neoplasm” OR “Malignancy” OR “Malignancies” OR “Malignant Neoplasms” OR “Neoplasm, Malignant” OR “Neoplasms, Malignant””**

**In Chinese：**

**((主题=穴位 OR 针灸 OR 体针 OR 针刺 OR 电针 OR 针*) AND 主题=癌症 OR 肿瘤 OR 癌*) AND 主题=加速康复外科* AND 文献类型=原创论文 AND 研究类型=(基于临床人群研究 OR 基于自然（一般）人群研究) AND 研究方法=随机对照试验**

**4 results**

**Supplementary studies were searched from references of include studies and clinical trial registration centers as follows:**

**a. http://www.chictr.org.cn**

**b. https://www.clinicaltrials.gov/**

**c. https://www.clinicaltrialsregister.eu/**
